# The state of diet-related NCD policies in Afghanistan, Bangladesh, Nepal, Pakistan, Tunisia and Vietnam: a comparative assessment that introduces a ‘policy cube’ approach

**DOI:** 10.1093/heapol/czz175

**Published:** 2020-02-24

**Authors:** Kent Buse, Wafa Aftab, Sadika Akhter, Linh Bui Phuong, Haroun Chemli, Minakshi Dahal, Anam Feroz, Sayad Hofiani, Nousheen Akber Pradhan, Iqbal Anwar, Hajer Aounallah Skhiri, Jalila El Ati, Kim Bao Giang, Mahesh Puri, Bashir Noormal, Fauziah Rabbani, Sarah Hawkes

**Affiliations:** 1 UNAIDS, Avenue Appia 20, 1211 Genève, Switzerland; 2 Department of Community Health Sciences, Aga Khan University, Stadium Road, P. O. Box 3500, Karachi 74800, Pakistan; 3 Health System and Population Studies Division, icddrb, GPO Box 128, Dhaka 1000, Bangladesh; 4 Center for Population Health Sciences, Hanoi University of Public Health, No. 1A, Duc Thang Street, Duc Thang Ward, Bac Tu Liem District, Hanoi City, Vietnam; 5 SURVEN (Nutrition Surveillance and Epidemiology in Tunisia) Research Laboratory, 11 Rue Jebel Lakhdar, Bab Saadoun, 1007, Tunis, Tunisia; 6 Center for Research on Environment, Health and Population Activities (CREHPA), P.O.Box. 9626, Kusunti (near Yatayat office), Lalitpur, Nepal; 7 Ministry of Public Health, Fifth Floor, Central Blood Bank Building, Cinema Pamir Area, Kabul, Afghanistan; 8 Faculty of Medicine of Tunis, University of Tunis El-Manar, Rue de la Faculte de Medecine, Tunis, Tunisia; 9 National Health Institute, Ministry of Health, 5/7 Rue El Khartoum, Diplomat, Bloc IV, 10ème étage, le Belvédère 1002 Tunis, Tunisia; 10 National Institute of Nutrition and Food Technology, SURVEN (Nutrition Surveillance and Epidemiology in Tunisia) Research Laboratory, 11 Rue Jebel Lakhdar, Bab Saadoun, 1007 Tunis, Tunisia; 11 Institute of Preventive Medicine and Public Health, Hanoi Medical University, No 1 Ton That Tung, Dong da District, Hanoi, Vietnam; 12 Institute for Global Health, University College London, 30 Guilford Street, London WC1N 1EH5, UK

**Keywords:** NCDs, policy analysis, accountability, authority, human rights, WHO Best Buys

## Abstract

We assessed the technical content of sugar, salt and trans-fats policies in six countries in relation to the World Health Organization ‘Best Buys’ guidelines for the prevention and control of non-communicable diseases (NCDs). National research teams identified policies and strategies related to promoting healthy diets and restricting unhealthy consumption, including national legislation, development plans and strategies and health sector-related policies and plans. We identified relevant text in relation to the issuing agency, overarching aims, goals, targets and timeframes, specific policy measures and actions, accountability systems, budgets, responsiveness to inequitable vulnerabilities across population groups (including gender) and human rights. We captured findings in a ‘policy cube’ incorporating three dimensions: policy comprehensiveness, political salience and effectiveness of means of implementation, and equity/rights. We compared diet-related NCD policies to human immunodeficiency virus policies in relation to rights, gender and health equity. All six countries have made high-level commitments to address NCDs, but dietary NCDs policies vary and tend to be underdeveloped in terms of the specificity of targets and means of achieving them. There is patchwork reference to internationally recognized, evidence-informed technical interventions and a tendency to focus on interventions that will encounter least resistance, e.g. behaviour change communication in contrast to addressing food reformulation, taxation, subsidies and promotion/marketing. Policies are frequently at the lower end of the authoritativeness spectrum and have few identified budgetary commitments or clear accountability mechanisms. Of concern is the limited recognition of equity and rights-based approaches. Healthy diet policies in these countries do not match the severity of the NCDs burden nor are they designed in such a way that government action will focus on the most critical dietary drivers and population groups at risk. We propose a series of recommendations to expand policy cubes in each of the countries by re-orienting diet-related policies so as to ensure healthy diets for all.

## Background: diet-related non-communicable diseases and Best Buy policy responses

Non-communicable diseases (NCDs) exert a high burden of disease globally—including through impact on premature mortality. Deaths from NCDs have risen over the past 30 years—driven in part by changes in age structures and population growth—with all countries suffering from an increasing burden of NCD-related deaths (GBD 2015 Mortality and Causes of Death Collaborators, 2016), which now account for over 70% of all deaths (NCD Countdown 2030 collaborators, 2018). Ezzati *et al.* (2018) and Hay *et al.* (2017) estimate that NCDs currently account for over 60% of the global burden of disability-adjusted life years (DALYs). Diet-related NCDs are significant contributors to the overall burden, with diets high in salt and low in fresh fruit/whole grains estimated to account for approximately half of deaths and two-thirds of diet-related DALYs (Ashkan *et al.*, 2019).

NCDs are associated with inequalities in exposure to their determinants—including on the basis of geographical location (Lancet Diabetes & Endocrinology, 2017), gender (Cortese and Ling, 2011), education (Williams *et al.*, 2018), poverty and socio-economic status (Barbeau *et al.*, 2004)—leading to inequities in distribution across societies. Low- and middle-income countries (LMICs) are particularly affected with higher age-standardized death rates than in high-income countries, including for cardiovascular diseases, diabetes and a number of other NCDs. A systematic review by Allen *et al.* (2017) of NCD risks in LMICs found clear differences in the distribution of risk behaviours including consumption of unhealthy fats, low consumption of fruit, vegetables and fish and diets high in processed foods. The authors found in general that less affluent groups ‘consum[e] the least healthy diet’, whereas more affluent groups consume more healthy foods while also consuming larger amounts of ‘fats, salt and processed food’ (Allen *et al.*, 2017).

Despite their prevalence, burden and inequitable distribution, NCDs have been neglected in global health policy discourse (absent from the Millennium Development Goals), funding (proportion of development assistance for health dedicated to NCDs has averaged between 1 and 2% of the total since 2000; Allen, 2017) and the NCD community has been described as being ‘semi-comatose’ (Horton, 2015). The Lancet Commission on Obesity (Swinburn *et al.*, 2019) called for ‘a radical rethink of business models, food systems, civil society involvement, and national and international governance to address’ the issue. Moreover, it is unclear the extent to which policy responses for NCD control have taken (in)equity and other public health concerns, such as rights and gender, into account.

We are finally beginning to see shifts towards greater prioritization and action on NCDs at global and national levels. The tide began to turn with the first UN General Assembly High-Level Meeting (HLM) on NCDs in 2011 and in 2015 an NCDs-related target was included in the Sustainable Development Goals (SDGs) agenda (target 3.4 to reduce premature mortality from NCDs by one-third by 2030). Yet, progress is slow with fewer than one in five countries currently on-track to reach the SDG NCD target (NCD Countdown 2030 collaborators, 2018). Countries increasingly recognize the importance of addressing NCDs and are seeking technical support to ensure robust policies. For example, in developing its 2018–19 Work Plan, World Health Organization (WHO) reported that countries placed NCDs as the top area for technical support on a list of 21 health topics (World Health Organization, 2017a). Nonetheless, to date, there has been little rigorous analysis of country-level policies to tackle many of the most prevalent NCDs associated with unhealthy diets.

In this article, we focus on policies addressing diet-related exposure associated with common NCDs (Friel *et al.*, 2013) in six countries—Afghanistan, Bangladesh, Nepal, Pakistan, Tunisia and Vietnam. The risks in our study include both under-consumption of ‘healthy’ foods (e.g. fresh vegetables and fruit) alongside over-consumption of unhealthy foods high in sugar, salt and trans-fats—particularly when associated with the consumption of ultra-processed and highly processed products (World Economic Forum and World Health Organization, 2011).

We compared policy content against the WHO evidence-informed ‘Best Buys’ and other WHO-recommended interventions to address NCDs. The concept of ‘Best Buys’ was put forward in a joint publication between WHO and the World Economic Forum in 2011 (World Economic Forum and World Health Organization, 2011; World Health Organization, 2013b) in preparation for the HLM on NCDs the same year. These are evidence-based and ranked according to ‘cost-effectiveness, feasibility and non-financial considerations’ (World Health Organization, 2013a,b). The Best Buys have been subject to a number of updates with the current iteration dated 2017 (World Health Organization, 2017b) and approved by the World Health Assembly (World Health Organization, 2017c) presented in three groups: (1) most cost-effective and feasible (<US$100/DALY averted), (2) those >$100/DALY averted and (3) recommended despite lacking the evidence of cost-effectiveness (Hyseni et al., 2017).

**Figure 1:**
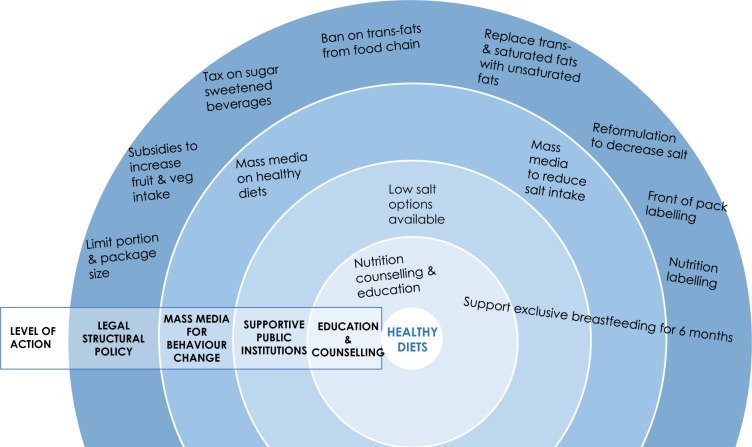
WHO Best Buys addressing unhealthy diet by level of action: societal, community, health services

The Best Buy interventions to address unhealthy diet are designed to act mainly on the structural drivers and commercial determinants of diet, an approach that is likely to yield greater benefits at the population level compared with individually focused interventions (Stuckler and Nestle, 2012; Roberts *et al.*, 2019; see Figure 1). Only a few of the Best Buys focus on individual- and community-level risks, with a small number to be delivered at the level of health services. Predominantly the WHO-recommended interventions are intended for implementation within and beyond the health sector, with policies and action required from, among others, the finance ministry, trade and industry ministry, taxation authority, food standard setting and regulatory agencies, media and advertising regulatory agencies, the education and agricultural sectors.

**Key Messages**

- Healthy diet policies in six countries do not match the severity of the NCDs burden nor are they designed in such a way that government action will focus on the most critical dietary drivers and population groups at risk.
- There is considerable scope for improving dietary NCD control policies in each of these countries—including by embracing the totality of World Health Organization dietary Best Buys, strengthening policy authoritativeness, identifying budgetary means, incorporating systems of accountability, promoting a greater focus on inequities in exposure across population groups and adopting human rights-based approaches.
- The policy cube introduced in this article provides a visual representation of the strength of dietary polies in each country; it could be adapted to visualize the strength of other public health policies.

There have previously been evaluations of the likely impact of Best Buys on NCD-related morbidity and mortality, including in LMICs (Allen *et al.*, 2018). However, there are fewer analyses focusing on the detailed content of existing national policies, or examination of their underlying principles to address equity, gender and rights, or the effectiveness of implementing each policy. In 2015 and 2017, WHO published member states’ updates on the extent to which 194 countries are implementing their commitments to develop national responses to the burden of NCDs. The four indicators addressing unhealthy diet assess whether or not a country has adopted policies to: (1) reduce salt consumption; (2) limit saturated fats and achieve virtual elimination of trans-fats; (3) adopt WHO recommendations on marketing of foods/drinks to children; and (4) implement the International Code of Marketing of Breast-Milk Substitutes (World Health Organization, 2015; World Health Organization, 2017d). These relatively non-specific indicators do not establish goals or policy actions—i.e. they are not as detailed as the Best Buys.

A five-country comparison of NCD policy processes in sub-Saharan countries noted that food-security and nutrition policies exist in almost all included countries, but Best Buy interventions for unhealthy diet had not received adequate attention except in South Africa (Juma *et al.*, 2018). This study and a review of Zambia’s National NCD Strategic Plan (Mukanu *et al.*, 2017) did not, however, report on policies in detail.

The six countries in our study represent a mix of low- and middle-income status; the proportion of the population living below the poverty line (US$1.19/day) ranges from 0.3% in Tunisia to 54% in Afghanistan, with a range of state approaches to governance, governmentality as well as regulation of the economy and health sector, and a mix of epidemiological profiles in relation to NCDs. The study aimed to examine the content and focus of NCD policies at national level and compare these with the WHO recommendations. The second objective was to evaluate the extent to which the national policies followed core attributes of effective (means of implementation) and equitable (including addressing human rights and gender) public policies to improve population health outcomes.

## Methods

**Figure 2:**
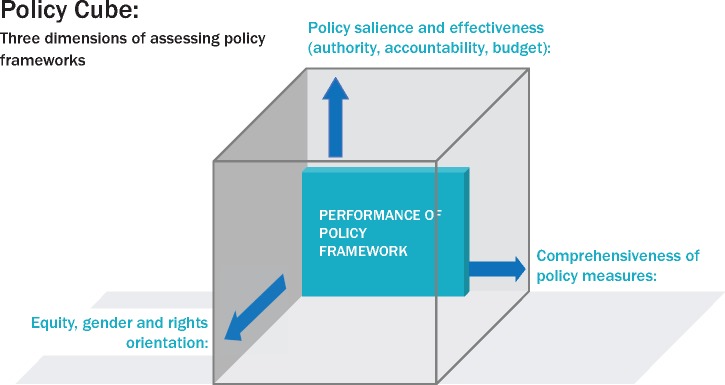
Three dimensions to assess the robustness of diet-related NCD frameworks: the policy cube approach

We were concerned with three dimensions of policy content. The first dimension is a quantitative assessment of how many WHO Best Buys were included in policy documents in each country. The second dimension refers to political salience and effectiveness of means of implementation, which included the presence of three components: presence of budget line item, level of policy authority and systems of accountability. The third dimension is the inclusion of key public health principles in the policy, i.e. recognition of human rights, addressing population health inequity including in relation to gender. We developed a graphic framework for bringing these dimensions together into a ‘policy cube’ (see Figure 2) that captures these three axes of policy content review and is discussed further in results.

### Comprehensiveness of policy coverage

We compared national documents against the 13 Best Buys. For three of the Best Buys—namely, ‘reduce salt intake through the reformulation of food products to contain less salt and the setting of target levels for the amount of salt in foods and meals’, ‘eliminate industrial trans-fats through the development of legislation to ban their use in the food chain’ and ‘reduce sugar consumption through effective taxation on sugar-sweetened beverages’—we deconstructed them into specific sub-components to identify the policy goal, target and actions separately. We did this in recognition that some countries may have an overall goal but no targets or specific actions. Thus, for the WHO salt Best Buy, we measured four separate components: (1) goal to decrease salt consumption; (2) reformulation of food products to decrease salt content (World Health Organization, 2017c); (3) establish a target for salt level in food (World Health Organization, 2017c); and (4) 30% reduction in salt consumption (World Health Organization, 2016). In total, we reviewed 13 Best Buys with a total of 19 sub-components to improve diet. For each of these 19 recommendations and their associated sub-components, we undertook textual content analysis to identify stated goal, targets, whether or not goals/targets were time-bound, measures and/or activities. In addition, we reviewed countervailing policies that contravene, subvert or impair the implementation of Best Buy measures—particularly in documents arising outside the health sector (e.g. trade policy, food subsidy policies).

Health policy is generally formulated by a range of institutions, most notably parliaments, but also by executives, ministries and local governments (Berlan *et al.*, 2014; Howlett, 2019). Therefore, our policy content analysis included all relevant government-issued documents (from 2000 to 2019, in any relevant language) that were in one way or another concerned with production, subsidization, promotion, regulation, taxation and consumption of sugar, salts and trans-fats—both inside and outside the health sector. Documents were identified by online searching of government and, where appropriate, development partner websites and cross-referenced through interviews with key informants. Search terms included Best Buys’ nomenclature (e.g. ‘advertising’, ‘breast milk’, ‘nutrition labelling’) along with expanded terminology for key products—e.g. ‘sodium chloride’, ‘monosaccharides’, ‘fructose’, ‘trans-unsaturated fatty acids’, ‘partially hydrogenated fats/oils’. Documents reviewed included: national legislation, national development policies, plans and strategies, national standards and guidelines governing salt, sugar and trans-fats in foods and beverages, national health sector plans and strategies, national NCDs policies and plans and reports and plans from regulatory agencies. Given the federal nature of policy-making in Pakistan (Nishtar *et al.*, 2013),^1^ we also analysed relevant documents from all four Provinces in the country.

### Political salience and effectiveness of means of implementation

Our study sought to assess the level of authority associated with each policy and interrogated evidence of other mechanisms to support implementation—i.e. the extent to which policies are explicit on systems of accountability, and the presence of an identified budgetary line item.

#### Policy authority

Not every policy document is of equal stature: a document that has been subject to national consultation, gone through a parliamentary committee and adopted by two houses of parliament has greater authority than a document drafted without inclusive processes and rubber-stamped by a ministerial technical committee. Authority is defined as having the legitimacy to influence, induce and/or enforce compliance and is largely related to the status of the government body issuing the policy (Klein and Marmor, 2006). Hence, we categorized relevant documents in relation to their relative ‘authoritativeness’—indicating the likelihood that bureaucrats, industry and society would act on them (Howlett, 2019). A country-focused hierarchy of the authoritativeness of policy documents was determined by each national team with the authority of each policy categorized as high, middle or low (see Table 1).

**Table 1:** Hierarchy of policy authority in each country

	Afghanistan	Bangladesh	Nepal	Pakistan	Tunisia	Vietnam
Highest level of authority	Constitution Act Laws Regulation (with presidential approval) Afghanistan national peace and development framework	Constitution Act Laws (legislation/ statue)	Constitution Act Laws/case law	Constitution Act (legislation/ statue)	Constitution International treaties Laws	Constitution Legislation Law Decree
Middle level of authority	Rules Regulations Policy Strategy National action plan	Rules Regulations Policy	Rules Regulations Policy	Rules Regulations Policy	Regulatory bloc By-laws Circulars	Rules Regulations
Lowest level of authority	Guidelines Standards Action plan Implementation plan	Guidelines Standards Yearly operational plans Strategy Activity Action plans Implementation plan	Directives Guidelines Strategy Action plans	Guidelines Action plans Standards Strategies	Contracts Conventions Standards Guidelines	Plans Strategies Guidelines Standards

#### Budget and systems of accountability

We assessed two further criteria: (1) the absence or presence of a stated budgetary line item to finance the policy measure and (2) the clear articulation of systems of accountability. We based measurements of accountability, a frequently contested concept (Brandsma and Schillemans, 2013; Pérez Durán, 2016), on key concepts of the accountability of public institutions to deliver public policy (Dubnick and Frederickson, 2009; Williams and Hunt, 2017). Systems of accountability within a policy were considered comprehensively addressed if all three of the following features were present and explicitly articulated: (1) a national lead/implementing agency is named and is assigned responsibility for reporting in the public domain; (2) a mechanism for independent monitoring of progress on implementation is described; and (3) remedial actions/sanctions/fines are outlined if implementation progress does not occur (Brandsma and Schillemans, 2013; Williams and Hunt, 2017).

### Principles of equity and rights

Finally, we investigated the extent to which these national NCD policies conform to WHO guidance on principles of rights-based, equity-focused approaches for improving health for all (World Health Organization, 2014). We interrogated the documents with two considerations: (1) given inherent inequitable distribution of NCDs and risk factors, plus disproportionate burdens and/or barriers some populations face in benefiting from policy actions we looked for acknowledgement of vulnerable/at-risk populations as a particular target or concern of the policy. While focused on equity broadly, we specifically looked for any acknowledgement of gender-related inequities; (2) we also investigated the articulation of principles of human rights in the policies (Hunt, 2016).

We were aware that the absence of language and consideration for equity, gender, and rights might reflect the status of health policy more generally in each country—i.e. these principles may not be missing only from diet-related NCD policies, but from other areas of health policy too. We therefore undertook a comparison with human immunodeficiency virus (HIV) prevention and control policies in each country so as to review whether or not the NCDs and HIV policies were equally concerned with equity and human rights obligations (Hunt, 2016).

### Our overall approach

Despite the benefits of policy analysis, including a deeper understanding of the context, politics and processes of prevailing health policy goals, as Gilson and Raphaely (2008) note, there is a relative dearth of capacity for undertaking health policy analysis within many LMIC. Therefore, one aspect of this study was the building of a cadre of young policy analyst researchers. Through regular training sessions from 2017 onwards, including in person, through joint activities, and involving regular group meetings, the researchers were trained in the key elements of undertaking health policy analysis (Buse *et al.*, 2012).

Research teams were drawn from a range of institutions, but all were nationally based. One team is based in the Ministry of Public Health (Afghanistan); two teams (Tunisia, Vietnam) work in institutions within or aligned to their respective ministries of health (MoH); in Bangladesh and Nepal, the research teams have close relationships with the MoH; and in Pakistan, the researchers are based in a university that works closely with the MoH. The quasi-embeddedness of many researchers was considered beneficial for the conduct of the project for four reasons: (1) these researchers expressed an interest in strengthening their policy analysis skills and would contribute to sustainable policy capacity in the sector; (2) the researchers have participated in or have access to NCDs-related policy-making and so had some familiarity with content and processes and access to actors involved; (3) as relative ‘insiders’, they provided credibility to the research endeavour; and (4) their positions would subsequently enable them to influence further development of evidence-informed NCDs policy as policy entrepreneurs (Walt *et al.*, 2008).

## Results

### Comprehensiveness of policy content

All countries in our study have recognized the importance of addressing unhealthy diets and have developed some level of corresponding policy. All countries have stated goals to decrease salt and sugar consumption but only three have adopted a goal to eliminate trans-fats. In Table 2, we list the presence and absence of Best Buys in each country, as well as the document(s) (with date) containing the policy. In Pakistan, all four provinces were found to have similar policy content and we therefore combined these into one unit of analysis. The one exception in Pakistan is in the case of marketing processed foods.

**Table 2:** Presence or absence of Best Buys and policy references in six countries

Level of effectiveness of recommendation	WHO recommendation	Sub-components of recommendation analysed in national policies	AF	BD	NP	PK—all four provinces	TN	VN
Best Buys: effective interventions with cost-effectiveness	Reduce salt intake through the reformulation of food products to contain less salt and the setting of target levels for the amount of salt in foods and meals	Goal to decrease salt consumption	NHP, 2015	MSAP, 2018	MSAP, 2014	Pakistan Dietary Guidelines, 2018	NSPCO	National Action Plan on Nutrition, 2018
		Reformulation of food products to decrease salt	NSNCDs, 2015	MSAP, 2018	MSAP, 2014	×	NSPCO	MoH Plan for NCD control, 2015
		Set target salt level in foods	×	×	MSAP, 2014	Punjab Pure Food Rules; Sindh Food Regulations	NSPCO	Vietnam Standards, 2004 and 2013
		30% reduction in salt consumption	×	MSAP, 2018	MSAP, 2014	×	NSPCO	National strategy on NCD control, 2015
	Reduce salt intake through the establishment of a supportive environment in public institutions, such as hospitals, schools, workplaces and nursing homes, to enable lower sodium options to be provided	Public institutions to have supportive environment	×	MSAP, 2018	MSAP, 2014	×	NMSNCDs	MoH Plan for NCD control, 2015
	Reduce salt intake through a behaviour change communication and mass media campaign	Mass media campaign to reduce salt intake	NHP, 2015	MSAP, 2018	MSAP, 2014	×	NMSNCDs NSPCO	MoH project for NCD communication and social mobilization, 2016
		Behaviour change communication on salt	×	MSAP, 2018	MSAP, 2014	×	NMSNCDs NSPCO	National strategy on NCD control, 2015
		Front-of-pack labelling	×	×	×	×	NSPCO	×
Effective interventions with CEA >/$100 per DALY averted in MICs	Eliminate industrial trans-fats through the development of legislation to ban their use in the food chain	Goal to eliminate industrial trans-fats	×	×	MSAP, 2014	×	NSPCO	× (Laws support importing trans-fat products)
		Legislation to ban the use of trans-fats in food chain	×	×	×	×	×	×
	Reduce sugar consumption through effective taxation on SSBs	Goal to reduce sugar intake	NHP, 2015	MSAP, 2018	MSAP, 2014	Pakistan Dietary Guidelines for Better Nutrition	NSPCO	MoH guidelines on rational nutrition, 2001; National strategy on NCD control, 2015
		Taxation on SSBs	NHP, 2015	MSAP, 2018	×	×	NSPCO	National strategy on NCD control, 2015; MoF proposed law on excise taxes, 2017
Other recommended interventions from WHO guidance (CEA not available)	Subsidies to increase the uptake of fruits and vegetables	Fruit and vegetable subsidies	NIP, 2018	×	MSAP, 2014	×	×	×
	Replace trans-fats and saturated fats with unsaturated fats through reformulation, labelling, fiscal policies or agricultural policies	Replace trans-fats and saturated fats with unsaturated fats through reformulation, labelling, fiscal policies or agricultural policies	×	MSAP, 2018	MSAP, 2014	×	NSPCO	×
	Limiting portion and package size to reduce energy intake and the risk of overweight/obesity	Limit portion and package size	×	×	×	Pakistan Dietary Guidelines for Better Nutrition	×	×
	Implement nutrition education and counselling in different settings (e.g. in preschools, schools, workplaces and hospitals) to increase the intake of fruits and vegetables	Public institutions promote fruit and vegetables consumption	×	MSAP, 2018	National nutrition policy, 2004	×	NMSNCDs	National Action Plan on Nutrition, 2018
	Implement nutrition labelling to reduce total energy intake (kcal), sugars, sodium and fats	Nutrition labelling	×	MSAP, 2018	MSAP, 2014	Pakistan Standards for Guidelines on Nutrition Labelling	NSPCO	MoH Plan for NCD control, 2015
	Implement mass media campaign on healthy diets, including social marketing to reduce the intake of total fat, saturated fats, sugars and salt, and promote the intake of fruits and vegetables	Mass media to promote healthy diet	NSNCDs, 2015; NHP, 2015	MSAP, 2018	National nutrition policy, 2004	×	NMSNCDs, NSPCO	National Action Plan on Nutrition, 2018
	Promote and support exclusive breastfeeding for the first 6 months of life, including promotion of breastfeeding	Promotion of exclusive breastfeeding	NRMNCHS, 2017–21 and RPSCFB, 2009	BMS Act, 2013	MMSA, 1992	Pakistan Infant and Young Child Feeding Strategy, 2016–20	NMSNCDs	Law on trading in and use of mother milk substitutes to protect and encourage the breast feeding, 2000

× = absence of policy.

AF: NHP = National Health Policy, 2015–20; NIP = National Implementation Plan of Afghanistan for NCDs and injuries, 2018–20; NSNCDs = National Strategy for Prevention and Control of NCDs, 2015–20; NRMNCHS = National Reproductive, Maternal, Newborn, Child and Adolescent Health (RMNCH) Strategy 2017–21; RPSCFB = Regulation on Protection and Strengthening of Child Feeding by Breast Milk, 2009.

BD: MSAP = Multisectoral Action Plan for Prevention and Control of Noncommunicable Diseases (2018–25), 2018, Ministry of Health & Family Welfare, Government of People’s Republic of Bangladesh; BMS = Breast-Milk Substitutes (BMS) Act, 2013. Government of People’s Republic of Bangladesh.

NP: MMSA = The Mother’s Milk Substitutes (Control of Sale & Distribution) Act, 1992; MSAP = multisectoral action plan for prevention and control of non-communicable diseases (2014–20), 2014, Government of Nepal; National Nutrition Policy and Strategy 2004, Ministry of Health and Population.

PK: Pakistan Dietary Guidelines for Better Nutrition, Government of Pakistan-Food and Agriculture Organization of the United Nations, 2018; Pakistan Standards for Guidelines on Nutrition Labelling (first revision), Pakistan Standards and Quality Control Authority PS, 2009–17; Pakistan Infant and Young Child Feeding Strategy, 16–2020; Punjab Pure Food Rules, 2011, The Government of Punjab Health Department; Sindh Food Authority Regulations, 2018, Food Department, Government of Sindh.

TN: NSPCO = national strategy to prevent and control obesity (2013–17); NMSNCDs = National Multisectoral Strategy to Prevent and Control of Noncommunicable Diseases 2018–25.

VN: plan for NCD control and prevention 2015–20 (issued in 2015), Ministry of Health, Vietnam; Vietnam standards for instant noodle (issued in 2004), Ministry of Science and Technology, Vietnam; Vietnam standards for cooked cured pork shoulder (issued in 2013), Ministry of Science and Technology, Vietnam; national strategy on NCD control and prevention in the period 2015–25 (issued in 2015), the Government of Vietnam; approval of project about communication and social mobilization in control and prevention of NCD from 2016 to 2020 (issued in 2016), Ministry of Health, Vietnam; guidelines on rational nutrition: 10 tips for proper nutrition for period (issued in 2001), Ministry of Health, Vietnam; proposed law on the amendment of excise taxes 2017: discussion on increase tax on non-alcoholic carbonated soft drinks 10% from 2019, Ministry of Finance, Vietnam; legislation regarding the trading in and use of mother milk substitutes to protect and encourage the breast feeding (issued in 2000), The Government of Vietnam.

AF, Afghanistan; BD, Bangladesh; NP, Nepal; PK, Pakistan; TN, Tunisia; VN, Vietnam; CEA = Cost-effectiveness analysis.

#### Highly cost-effective interventions

Four of the 13 dietary Best Buys are considered highly cost-effective by WHO, and all four of these pertain to limiting salt consumption (Hyseni *et al.*, 2017). All countries have adopted a goal for salt reduction, with a time-bound target of 30% reduction by 2025 in Bangladesh, Nepal and Vietnam. Tunisia targets a 30% reduction, but this does not appear to be time-bound. Pakistan has a goal of salt reduction but no clear targets or time frames. All countries mention at least one policy measure to reduce salt consumption—reformulation, supportive environments (meals and food in schools and other public institutions) and behaviour change campaigns. Only one of the countries (Tunisia) has a policy on front-of-pack labelling for reducing salt consumption.

#### Less cost-effective interventions

These two Best Buys concern industrial trans-fats and sugar. All countries have a goal to reduce sugar consumption, whereas only two countries (Tunisia and Vietnam) have a goal to reduce trans-fats. Four countries (Afghanistan, Bangladesh, Tunisia and Vietnam) have signalled their intention to introduce taxes on sugar-sweetened beverages (SSBs). None of the six countries has mentioned introducing legislation to ban the use of trans-fats in the food chain, and Vietnam has a policy supporting the import of trans-fats.

#### Other recommended interventions

The other six recommended policy measures cover salt, sugar and trans-fats as well as promotion of a healthy diet and breastfeeding. Five countries (not Afghanistan) have policies on nutritional labelling. Afghanistan and Nepal briefly mention agricultural subsidies to promote the consumption of fresh fruit and vegetables. Nonetheless, all countries have policies in place that seek to ensure the security of the food supply.

In a number of countries, policies were identified which could be considered inimical to NCD-related public health goals as they promote the production and/or subsidize consumption of health-harming foods. For example, in Nepal, Pakistan and Tunisia, governments provide subsidies for sugar and, in Bangladesh, biscuits are subsidized (see Table 3). Food subsidies are generally targeted at the poor, or people with other structural vulnerabilities—e.g. living in remote districts (Nepal), or members of ethnic minority communities (Vietnam). No country was found to subsidize access to healthy fruit and vegetables; however, Tunisia provides a subsidy for industrial tomato products (for the harissa condiment). In Vietnam, a number of trade and import policies are targeted on protecting the domestic sugar industry with support to cane growers and sugar mill factories (World Trade Organization, 2006) and the local food processing industry in its production ‘of candy, cake, instant noodle’ (Government of Vietnam, 2013).

**Table 3:** Food and agricultural subsidies and target populations

	Food/goods subsidized	Type of subsidy	Target population or beneficiaries
Afghanistan	Flour	Price subsidy for production	Mills, nationwide
	Iodized salt	Price subsidy	Nationwide
Bangladesh	Rice/wheat	Price subsidy	Poor households
	Rice/flour	Food ration	Poor and vulnerable women
	Rice/wheat	Food ration	Poor households in disaster-struck areas
	Biscuits	Free fortified biscuits	School children
	Rice/wheat	Subsidized price	Poor people
	Rice/wheat	Food as wages	Rural women
Nepal	Rice/wheat/buckwheat/beans	Price subsidy	Nationwide during festivals
	Salt, sugar, rice, ghee, oil, lentils, flour, beaten rice	Price subsidy	Nationwide during festivals
	Seeds/fertilizer	Price subsidy	
	Salt, rice, wheat, buckwheat, beans	Transport subsidy	People in remote districts
	Rice	Ration of 20 kg	Family events in one remote district
Pakistan	Wheat	Price purchase subsidy	Farmers and poor people
	Oil, sugar, dates, flour and pulses	Price subsidy	Nationwide during Ramadan
	Sugar	Export subsidy	Sugar producers
Tunisia	Semolina, couscous, pasta flour, bread, industrial tomato, milk, sugar, vegetable oil	Price subsidy ranging from 4.2% (industrial tomato) to 56.3% (semolina)	Protect purchasing power of poor Tunisians, guarantee of a minimum income for cereal farmers
Vietnam	Rice	Rations	Poor households
	Rice	Farming support	Ethnic minority households

### Policy authoritativeness

Each country team identified laws and statutes as carrying the highest level of authority, followed by rules and regulations, with standards, guidelines, strategies and action plans at the lowest level of policy authoritativeness (Table 1). Policies concerned with dietary NCDs tended to be at the lower end of authoritativeness spectrum in most of the countries except Tunisia (see Table 4). In Afghanistan, Bangladesh, Nepal and Pakistan, there are high authority policies relating to the restriction of marketing of complementary foods to infants (and promoting exclusive breastfeeding); Nepal also has one high authority policy addressing mass media campaigns. In contrast, most policy responses in Tunisia are considered to be high authority—14 of the 16 Best Buy sub-components present are judged to be high authority. For Vietnam, the picture is more mixed—there are 13 policy sub-components and six are high authority.

**Table 4:** Assessments of Au, Ac and Bu for Best Buys in national policies for diet-related NCD control

WHO recommendation by the level of effectiveness	Best Buy policy recommendations from WHO	Sub-components of recommendation analysed in national policies	Countries					
			AF	BD	NP	PK	TN	VN
Best Buys: effective interventions with cost-effectiveness	Reduce salt intake through the reformulation of food products to contain less salt and the setting of target levels for the amount of salt in foods and meals	Goal to decrease salt consumption	Au = ▲ Ac = ◼ Bu = ◼	Au = ◼ Ac = ▲ Bu = ▲	Au =◼ Ac = ▲ Bu = ◼	Au = ◼ Ac = ◼ Bu = ◼	Au = ● Ac = ◼ Bu = ◼	Au = ▲ Ac = ▲ Bu = ●
		Reformulation of food products to decrease salt	Au = ▲ Ac = ▲ Bu = ◼	Au = ◼ Ac = ▲ Bu = ◼	Au = ◼ Ac = ▲ Bu = ◼	**×**	Au= ● Ac = ● Bu = ●	Au = ● Ac = ▲ Bu = ●
		Set a target salt level in processed foods	**×**	**×**	Au = ◼ Ac = ▲ Bu = ◼	Au = ▲ Ac = ▲ Bu = ◼	Au = ● Ac = ● Bu = ●	Au = ● Ac = ▲ Bu = ●
		Overall target of 30% reduction	**×**	Au = ◼ Ac = ▲ Bu = ▲	Au = ◼ Ac = ▲ Bu = ◼	**×**	Au = ● Ac = ◼ Bu = ◼	Au = ▲ Ac = ▲ Bu = ●
	Reduce salt intake through the establishment of a supportive environment in public institutions, such as hospitals, schools, workplaces and nursing homes, to enable lower sodium options to be provided	Public institutions to have supportive environment	**×**	Au = ◼ Ac = ▲ Bu = ▲	Au = ◼ Ac = ▲ Bu = ◼	**×**	Au = ● Ac = ▲ Bu = ▲	Au = ▲ Ac = ▲ Bu = ▲
	Reduce salt intake through a behaviour change communication and mass media campaign	Mass media to reduce salt	Au = ▲ Ac = ◼ Bu = ◼	Au = ◼ Ac = ▲ Bu = ●	Au = ◼ Ac = ▲ Bu = ●	**×**	Au = ● Ac =◼ Bu = ●	Au = ● Ac = ● Bu = ●
		Behaviour change communication to reduce salt	**×**	Au = ◼ Ac = ▲ Bu = ●	Au = ◼ Ac = ▲ Bu = ●	**×**	Au = ● Ac = ◼ Bu = ●	Au = ● AC = ● Bu = ●
	Reduce salt intake through the implementation of front-of-pack labelling	Front-of-pack labelling	**×**	**×**	**×**	**×**	Au = ● Ac = ● Bu = ●	**×**
Effective interventions with CEA >/$100 per DALY averted in LMICs	Eliminate industrial trans-fats through the development of legislation to ban their use in the food chain	Eliminate industrial trans-fats	**×**	**×**	Au = ◼ Ac = ▲ Bu = ◼	**×**	Au = ● Ac = ◼ Bu = ◼	Au = ◼ Ac = ◼ Bu = ◼
		Legislation to ban use of trans-fats in food chain	**×**	**×**	**×**	**×**	**×**	**×**
	Reduce sugar consumption through effective taxation on SSBs	Goal to reduce sugar intake	Au = ▲ Ac = ◼ Bu = ◼	Au = ◼ Ac = ▲ Bu = ▲	Au = ◼ Ac = ▲ Bu = ◼	Au = ◼ Ac = ◼ Bu = ◼	Au = ● Ac = ◼ Bu = ●	Au = ▲ Ac = ◼ Bu = ◼
		Taxation on SSBs	Au = ▲ Ac = ◼ Bu = ◼	Au = ◼ Ac = ▲ Bu = ◼	**×**	**×**	Au = ● Ac = ● Bu = ●	Au = ▲ Ac = ▲ Bu = ▲
Other recommended interventions from WHO guidance (CEA not available)	Implement subsidies to increase the intake of fruits and vegetables	Subsidies for fruit and vegetables	Au = ◼ Ac = ◼ Bu = ◼	**×**	Au = ◼ Ac = ▲ Bu = ◼	**×**	**×**	**×**
	Replace trans-fats and saturated fats with unsaturated fats through reformulation, labelling, fiscal policies or agricultural policies	Replace trans-fats and saturated fats with unsaturated fats through reformulation, labelling, fiscal policies or agricultural policies	**×**	Au = ◼ Ac = ▲ Bu = ◼	Au = ◼ Ac = ▲ Bu = ◼	**×**	Au = ● Ac = ▲ Bu = ◼	**×**
	Limiting portion and package size to reduce energy intake and the risk of overweight/obesity	Limit portion size	**×**	**×**	**×**	Au = ◼ Ac = ◼ Bu = ◼	**×**	**×**
	Implement nutrition education and counselling in different settings (e.g. in preschools, schools, workplaces and hospitals) to increase the intake of fruits and vegetables	Public institutions promote fruit and vegetables consumption	**×**	Au = ◼ Ac = ▲ Bu = ●	Au = ▲ Ac = ▲ Bu = ●	**×**	Au = ● Ac = ◼ Bu = ◼	Au = ● Ac = ● Bu = ●
	Implement nutrition labelling to reduce total energy intake (kcal), sugars, sodium and fats	Implement nutrition labelling to reduce total energy intake (kcal), sugars, sodium and fats	**×**	Au = ◼ Ac = ▲ Bu = ◼	Au = ◼ Ac = ▲ Bu = ◼	Au = ◼ Ac = ▲ Bu = ◼	Au = ◼ Ac = ● Bu = ●	Au =▲ Ac = ▲ Bu = ◼
	Implement mass media campaign on healthy diets, including social marketing to reduce the intake of total fat, saturated fats, sugars and salt, and promote the intake of fruits and vegetables	Mass media to promote healthy diets	Au = ▲ Ac = ▲ Bu = ◼	Au= ◼ Ac = ▲ Bu = ◼	Au = ▲ Ac = ▲ Bu = ●	**×**	Au = ● Ac = ◼ Bu = ◼	Au = ▲ Ac = ▲ Bu = ▲
	Promote exclusive breastfeeding	Promote exclusive breastfeeding	Au = ● Ac = ▲ Bu = ◼	Au = ● Ac = ▲ Ac = ●	Au = ● Ac = ▲ Ac = ●	Au = ● Ac = ◼ Bu = ◼	Au = ● Ac = ● Bu = ●	Au = ● Ac = ● Bu = ●

×, no policy found.

Au: ● = high authority (see Table 1 for full details); ▲ = medium authority; ◼ = low authority.

Ac: ● = abides by key principles of accountability, namely: (1) a national lead/implementing agency is named and is assigned responsibility for reporting in the public domain; (2) a mechanism for independent monitoring of progress on implementation is described; and (3) remedial actions/sanctions/fines are outlined if implementation progress does not occur; ▲ = a national lead/implementing agency is named and is assigned responsibility for reporting in the public domain; ◼ = no mechanism for accountability found.

Bu: ● = budget line item assigned to policy sub-component; ▲ = budget line item planned but no evidence for line item identified; ◼ = no budget line item identified.

AF, Afghanistan; BD, Bangladesh; NP, Nepal; PK, Pakistan; TN, Tunisia; VN, Vietnam; Au, authority; Ac, accountability; Bu = budget; MoF = Ministry of Finance; CEA = Cost-effectiveness analysis.

### Accountability

Systems of accountability for the Best Buys in Bangladesh and Nepal are most frequently limited to the identification of the agency responsible for implementation and reporting (see Table 4). Although both countries do promote the establishment of a national multisectoral co-ordination or steering committee, chaired by the Ministry of Health and Family Welfare (Bangladesh) or Chief Secretary to the Cabinet (Nepal). In Tunisia and Vietnam, there is a named agency and commitment to publicly available reporting for a number of the Best Buys (five Best Buys and three sub-components). Independent monitoring or remedial action for non-implementation was generally absent.

### Budget

Tunisia and Vietnam are most likely to have budgetary line items to support the implementation of the Best Buys (Table 4). Bangladesh and Nepal have specified budgets for mass media and education campaigns to reduce salt consumption and for the promotion of exclusive breastfeeding. In addition, Bangladesh has a line item for nutrition education in public institutions. Pakistan does not identify dedicated budgets for any of the specific Best Buys, but, along with Nepal and Bangladesh, it does have a line item for NCDs in the overall health budget.

### Health equity

Five countries (not Afghanistan) have identified children as a target group for NCDs prevention and control with a focus on school children in four countries (Nepal, Pakistan, Tunisia and Vietnam) (see Table 5). Likewise, all countries identify additional specific population groups for policy attention—such as the poor (Bangladesh), urban population (Afghanistan and Bangladesh) or people with heart disease or hypertension (Nepal, Tunisia and Vietnam). Beyond a general mention of gender as a social determinant of health in the multisectoral action plans of Nepal and Bangladesh, we did not find specific mention in policies of the impact of gender inequality, gender norms and gender roles on patterns of exposure to unhealthy food stuffs including, e.g. through gendered patterns of consumer purchasing or food preparation.

**Table 5:** HIV and NCD policies with mention of target population combined across six countries

HIV policies across six countries	Diet-related NCD policies across six countries
Male migrant workers Street children Gay men External migrants Mobile populations Internally displaced persons High risk women Female sex workers Hijra (transgender people) Male sex workers Transport workers Men who have sex with men People who inject drugs Prisoners Incarcerated people Uniformed forces Clients of sex workers Transgender sex workers Adolescents Persons with disabilities Long distance truckers Refugees Children	Hypertensive patients Patients with chronic heart diseases Patients with diabetes Elderly Children Office and factory workers School and college students, teachers and staff Urban population

In comparison to the relative dearth of targeted policies focused on specific population groups for diet-related NCD control, the HIV policies in all countries mention specific population sub-groups for focused attention (see Table 5).

### Human rights-based approaches

All countries had extensive and specific reference to human rights principles in their HIV policies (see Buse *et al.*, 2019a for further details), but only Bangladesh included rights-based language in dietary NCD policies (see Table 6).

**Table 6:** Comparison of mention of human rights in HIV and NCDs policies in Bangladesh

	Mention of human rights in HIV policies	Mention of human rights in NCDs policies
Bangladesh	*Necessity of enabling environment to address human rights violations (e.g. harassment of MARPs) and gender inequality (e.g. disempowerment of women in negotiation of safe sex) was identified. In this regard, partnership with Human Rights Commission to address HIV related violation of human rights was deemed suitable* (3rd National Strategic Plan for HIV and AIDS Response 2011–15). *This policy projects that by the year 2010, priority groups of people at risk of HIV infection will have access to the means of protection in ways that respect their human rights and dignity and will be empowered to protect themselves and others.* *It also strategies to reduce the vulnerability of children and young people living with and affected by HIV. In this purpose, protection of human rights and dignity of affected children and youth would be ensured. access to counselling and health services will be one of its focus* (2nd National Strategic Plan for HIV/AIDS 2004–10).	*Nutrition is a basic human right with both equity and equality related to eliminating malnutrition and ensuring human development* (National Nutrition Policy, 2015).

### Summarizing the findings for each country in a policy cube

In Figure 3, we apply the policy cube approach to illustrate the overall findings in each country. Each cube pictorially represents the extent to which the country’s NCD policy framework meets criteria assessed in our review—policy comprehensiveness (i.e. how many of the Best Buys were found in the policy documents of each country—data in Table 4), effective means of implementation (i.e. scoring levels of policy authority, systems of accountability and presence of a budget line item—data in Table 4) and equitable rights-based approaches (Tables 5 and 6). For example, in Nepal, we found 12 of the 19 WHO recommendations in national strategy and policy documents and have reflected that on the *x*-axis as adequately comprehensive. In contrast, relevant Nepalese documents on NCDs enjoy relatively low authority and do not identify accountability mechanisms nor budget sources and, hence, a low effectiveness is recorded on the *y*-axis. Given the low (frequently absent) attention paid to gender, equity and rights in Nepalese NCDs policies, the cube does not progress very far on the *z*-axis, which is concerned with equity. It should be noted that these cubes do not take into account any countervailing policies, which would act to further undermine the NCD policy intentions and shrink the policy cube.

**Figures 3:**
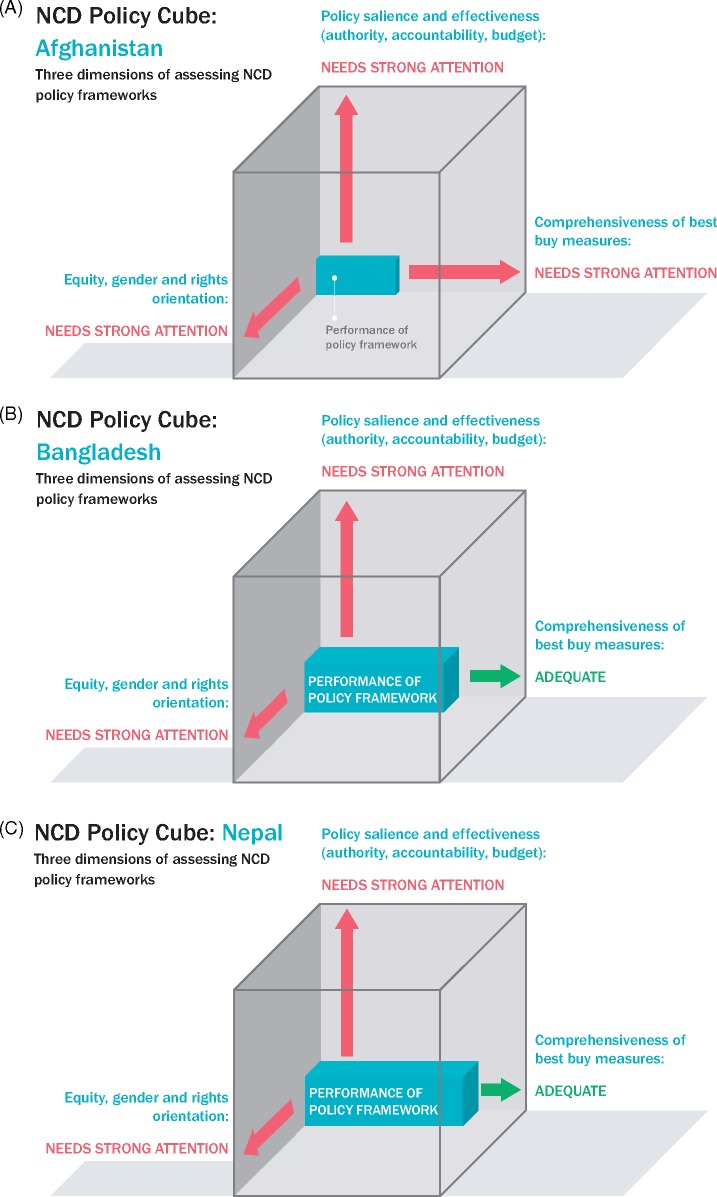
Policy cube results for each country. (**A**) Afghanistan. (**B**) Bangladesh. (**C**) Nepal. (**D**) Vietnam. (**E**) Pakistan. (**F**) Tunisia.

**Figures 3:**
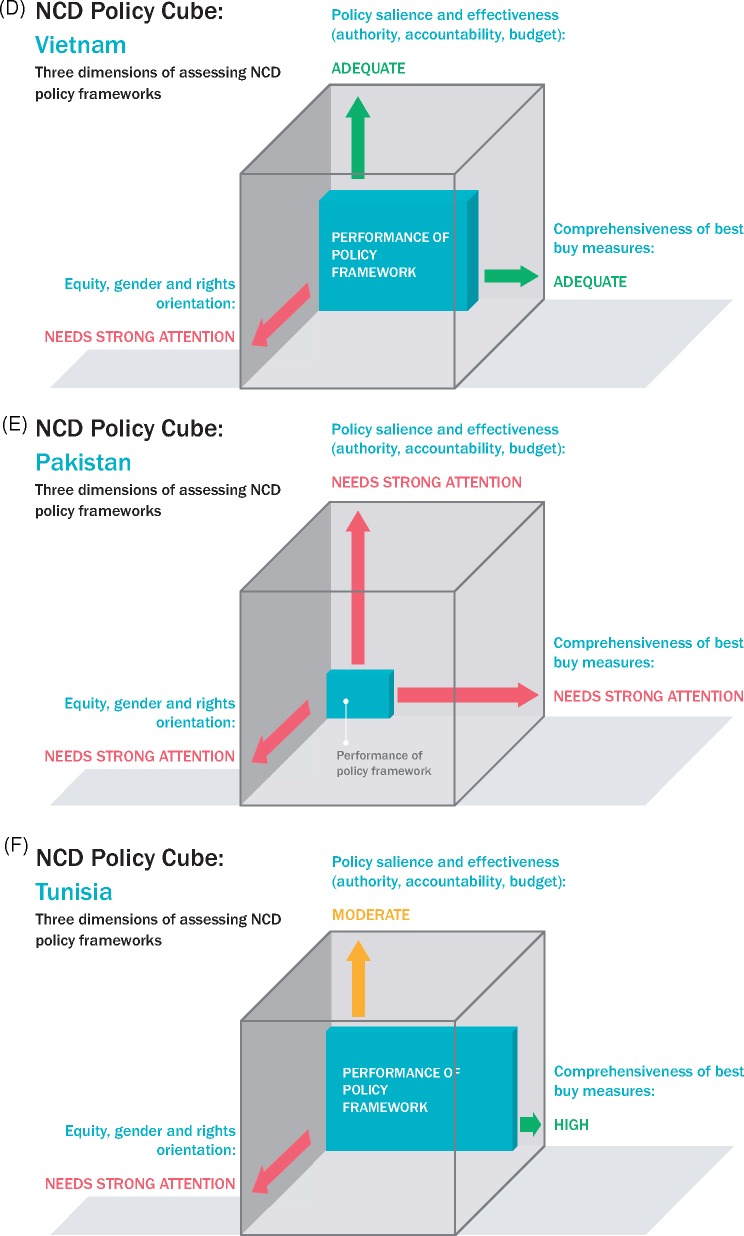
(continued)

## Discussion

The power of comparative analysis lies in its ability to make generalizations and potentially differentiate general factors from context-specific ones (Yin, 2014). This multi-country comparison of the aspirations for and means to address the risk factors for NCDs in six countries has highlighted a wide variation in the specificity, authority and likelihood of implementation of effective dietary policies to improve population health in relation to NCDs. While all six countries have recognized and initiated policy responses to the growing epidemic of NCDs, the adequacy, effectiveness, respect for principles of human rights and likely equity impact of these responses to tackle unhealthy diets must be called into question.

This analysis enabled us to assess the relative position of policies in terms of government prioritization (i.e. political salience of the policy) as well as the who, how, why and when of their potential impact on different interests and hence the likelihood of their implementation and impact (Kingdon, 2003). We sought to understand the policy aims and means of implementation (or lack thereof) as outlined in a range of government documents both inside and outside the health sector. Recognizing that for policies to be effective, they would have to move beyond content, to also incorporate key principles of public health policy, we assessed comprehensiveness, means of implementation and orientation towards addressing equity, rights and gender.

Nonetheless, content of the relevant text does more than guide government as it also provides a means to hold governments (or their departments) to account for commitments made (Brandsma and Schillemans, 2013). Content analysis can also help to reveal stakeholder positions (Dreser *et al.*, 2012; Karn *et al.*, 2017), but a broader approach to analysing stakeholders’ power and positions and context is needed to understand the role of power and interests in driving policy agendas—and is currently underway by the research teams in our study countries.

### Means of effective implementation: policy authority and political salience

A number of the Best Buy measures require policy measures at the level of laws, acts or decrees. This is self-evident in the case of Best Buy on the taxation of SSBs—but arguably mandatory laws are needed for non-voluntary action by industry in relation to food reformulation (Reeve and Magnusson, 2015), limits to portion size (Zlatevska and Spence, 2016), nutritional labelling (Magnusson, 2010; Hiilamo and Glantz, 2015) and reduced marketing of processed food (Boyland and Harris, 2017), while legislation would likely also be required for agricultural subsidies to promote the consumption of fruits and vegetables (Buse *et al.*, 2017). Nonetheless, across five countries (except Tunisia), the policy measures for interventions were to be found in less authoritative policy documents. For example, while both Bangladesh and Nepal had policies addressing most of the 13 Best Buys, these were all contained within a multisectoral action plan and not accompanied by legislative or regulatory support.

The majority of the WHO Best Buys to control unhealthy diet is focused on addressing structural determinants of risk (Figure 1), and we found a mixed picture in relation to policies operating in the structural domain. Governments in our study have tended to develop policies that could be considered ‘low hanging fruit’ and/or face least opposition. Consequently, we see more attention to promoting ‘supportive environments’ in public institutions (four countries), implementing behaviour change communications and mass media campaigns (four countries) and nutritional labelling (five countries) rather than front-of-pack labelling (no countries). This confirms the findings of Baum and Fisher (2014) who note that ‘behavioural approaches and policies have strong appeal to governments’ in contrast to addressing the social, structural and commercial environments that predominantly drive poor population health outcomes.

### Accountability and financing for implementation

Our detailed assessment highlighted the relative absence of systems of accountability and budgetary allocations to ensure implementation. We only found commitments to full systems of accountability—identification of a lead agency, independent reporting on progress towards achieving the Best Buys, putting that information in the public domain and the possibility of remedial action—across 6 of the 19 policy measures in Tunisia and four in Vietnam. In the other countries, there was sometimes mention of a lead agency responsible for the policy but no description of independent monitoring or other elements of accountability including remedial action (Williams and Hunt, 2017).The absence of policy mechanisms to promote implementation (including budgets) may indicate a relative lack of policy priority and/or lower likelihood of success—particularly if there are inadequate or absent mechanisms to hold government agencies to account for the non-delivery of policy aims (Williams and Hunt, 2017).

### Equity and rights

Most countries had a high-level commitment to the right to health in national constitutions, but in the case of diet-related NCDs, this did not translate into specific policy commitments based on considerations of equity and clear articulation of rights. The burden of diet-related NCDs falls differentially across population groups, and different population groups face different NCD risks—e.g. diabetes rates in urban populations can be higher than those found in their rural peers (Alam *et al.*, 2011; Dagenais *et al.*, 2016), and the urban poor are particularly vulnerable to the impact of NCDs, such as catastrophic household health expenditures (Vu *et al.*, 2016). We also know that the structural policy responses impact different population groups in different ways (Backholer *et al.*, 2016). NCDs policies ought to take into account which population groups are most at risk and the fiscal and public health impact of proposed policies on those groups. However, our analysis found both a lack of consistent mention of populations likely to be at risk of NCDs or likely to suffer more from their impact and a virtual absence of policies to protect vulnerable populations, including on the basis of gender, or subsidize access to healthier foods for those unable to afford them. Moreover, only one country (Bangladesh) gave reference to human rights in their overall NCD strategy—and this was in relation to ‘eliminating malnutrition and ensuring human development’ (Government of Bangladesh, 2018).

In contrast, HIV policies were more likely to make mention of the different groups at risk of infection and to promote strategies embedded in principles of human rights. It has been argued that the tremendous progress in the response to the HIV epidemic is in part a result of its rights-based orientation and actions (Buse *et al.*, 2019b). Among other things, the response asserted the rights of marginalized and vulnerable populations, educated people living with and affected by HIV of their rights so as to help them claim them, as well as developed legally binding principles describing State obligations in response to social justice and human rights challenges (United Nations, 2006). The articulation of such obligations provides a powerful tool for civil society organizations to hold States accountable for their responsibility to take actions to respect, protect and fulfil the right to health. While recognizing the fundamental differences between the two epidemics (HIV and NCDs), we nonetheless propose that the NCDs community can and should be making use of human rights arguments to progress more urgent action (Patterson *et al.*, 2019)—not least since it has been previously noted that ‘if social inclusion and human rights do not underpin policy formation, it is unlikely they will be inculcated in service delivery’ (MacLachlan *et al.*, 2012). This position is gaining a great deal of support (Buse *et al.*, 2019b). Our study confirms that in contrast to the HIV response, dietary NCD policies in the six countries are not sufficiently drawing on this powerful public health tool.

### Countervailing and conflicting policy measures

Our analysis has highlighted the presence of government policies that may act to perpetuate or enhance unhealthy diets. For example, agricultural subsidy policies that were presumably designed for situations of under-nutrition may require an additional approach in an era when over-nutrition is rising. While subsidies to promote access to cereals may still be needed for many vulnerable populations in the six countries (Table 3), state subsidies for sugar production and consumption should be called into question from a public health perspective. Moreover, we are concerned both about the absence of policies to promote the access/consumption of healthy foods including fruit and vegetables and the presence of policies to support the production of unhealthy foodstuffs—e.g. as found in Vietnam.

### Policies with high authority and widespread support

All countries had policy measures to meet the goal of promoting exclusive breastfeeding, and for five countries, this was at the highest level of authority. This finding may bear important lessons for NCD control more generally. Promotion of exclusive breastfeeding was not originally designed as a measure for NCD control, but as a measure of promoting child survival and infant health. However, it is included in the NCD Best Buys as there is increasing evidence that breastfeeding may be associated with ‘lower consumption of sweetened beverages or foods’ (Passanha *et al.*, 2018).

## Limitations

There are a number of methodological limitations that might impact on the validity of our findings. First, the searches may not have been exhaustive and we might have missed relevant policy documents, particularly if they were not publicly available. Second, the researcher interpretation of presence or absence of policy features may suffer from subjectivity—which was not fully minimized with triangulation—as might their assessments of authoritativeness of documents, particularly in those documents that may have lacked specific information to make clear judgements. Third, although we used comparative cross-policy analysis approach with the aim of increasing the likelihood that our findings may be generalizable (Yin, 2014) to other settings similar to the countries in our sample (Carter and Little, 2007), nonetheless, caution should be exercised in over-reaching generalizability in the interpretation of common findings (Fukuda-Parr *et al.*, 2013). Finally, a policy content analysis by definition only examines words on paper. The implementation of those policies may or may not follow, for any number of reasons, and it was not our intention to assess implementation at this stage of the project. The second phase of this study on policy context, process and stakeholder power analysis provides a more comprehensive analysis to understand those dynamics.

## Conclusions

Our study has taken an objective-rationalist^2^ approach to reviewing the content of diet-related NCDs policy against empirically derived technical guidance. In practice, the making of policy is less technical and more political in nature as it impacts on the interests of a range of actors, e.g. through the regulation of the market or the allocation of state resources. This is most obvious in economic policy (taxes, subsidies, etc.), but it is equally true in public health policy. According to Kingdon (2003), policy-making involves a series of authoritative choices between specified alternatives. Those alternatives include which bodies will be involved in policy formulation as well as alternatives in relation to goals and means. The choices will be a matter of political calculations based among other things on potential substantive impact on the social problem, how the policy measures respond or align to societal values and how they will be viewed by interest and groups and the public from whom governments seek political support and compliance for their policies. Viewed from this perspective, it would appear rational that governments would tend towards the adoption of non-binding policies that do not threaten interests and are easiest to implement.

Nonetheless, from a public health perspective, the finding that the majority of policies addressing the dietary Best Buys is strong on goals but generally tends to lack the characteristics and level of salience to ensure effective implementation, promote equity and uphold human rights, should be of concern. The authoritativeness of the breast-milk substitutes policy attests to the need for strong engagement of a transnational advocacy coalition with civil society engagement in support of national policy implementation. The burden of NCDs is rising, seemingly inexorably, in all countries—irrespective of stage of economic development—and inaction to address the unhealthy food environment is not an option. We have identified a set of actions (see Panel 1) that all countries should consider for ensuring that Best Buys policies are not only technically sound but also implemented—with the highest level of policy authority, clear mechanisms of accountability and ensuring that human rights in relation to healthy diets are respected, protected and fulfilled. Our analysis suggests that dietary NCD policies need to move from aspirational statements of intent to specific actionable measures. Implementing these measures will require adopting a more politically informed approach to understanding the interests of stakeholders affected by WHO Best Buys.

Finally, in this article, we introduced the Policy Cube as a visual aid to reflect the strength of the dietary policy framework and its likely impact on health equity outcomes. Each of the countries currently has a differently shaped and sized cuboid—but all could be expanded by enhancing elements of policy content. The cubes illustrate where more effort is needed. We suggest that the Policy Cube is a valuable instrument for assessing the strength and likely public health impact of other health policies.

## Panel 1—recommendations towards more effective and equity-oriented evidence-informed diet-related NCDs policy

1. Comprehensive policies. Countries should adopt holistic policy frameworks for the prevention of dietary NCDs that covers the breadth of WHO Best Buys and other technical guidance—particularly addressing areas that our analysis suggests might otherwise not be included including legislation to ban trans-fats, front-of-pack labelling and agricultural subsidies for fruits and vegetables targeted at both farmers and consumers.
2. Countervailing and conflicting policy measures. Members of NCD co-ordination platforms should ensure that trade, investment and other social policies do not inadvertently undermine the dietary-related public health policies, such as subsidies for sugar growers.
3. SMART targets. Countries should articulate dietary policy in relation to outcome goals, identify specific measures, set time-bound targets and guide action with accountability mechanisms outlined including independent monitoring of implementation.
4. Authoritative policy guidance. Countries are encouraged to embed these Best Buys interventions in legislation or other authoritative policy documentations, rather than action plans, to ensure a better probability of implementation.
5. Rights based. Evidence-informed NCDs policies should be grounded in a rights-based approach that recognizes both the right to a healthy food environment and the right to the management of illness—i.e. the right to keeping people healthy as well as care when sick.
6. Targeting most at-risk and vulnerable groups. Stakeholders should ensure policies include provisions to target efforts and resources at those populations most at risk and carrying a disproportionate burden of NCDS.
7. Resource allocation. Countries ought to ensure that policies identify adequate budgetary sources for ‘implementation’.
8. International technical support. Given the complex, political and contested nature of dietary-related policies, countries require international technical and strategic support to embed the evidence-informed Best Buys in policy, akin to the support they have received from partners, such as the Tobacco Framework Convention Alliance and Bloomberg Philanthropies for tobacco control policy.
9. Civil society and advocacy coalitions. International partners ought to support civil society, particularly consumer rights organizations, including to support their co-ordination to play a more active role in advocacy, evidence-informed policy development of healthy diet-related policies and robust accountability mechanisms.
10. Strengthening capacity for policy analysis. Public health practitioners and researchers who have an embedded understanding of policy context should be encouraged and supported to undertake policy content analysis to better identify challenges and opportunities for the implementation of evidence-informed recommendations.
11. Policy Cube. The cube provides a visual representation of the strength of the policy framework in any particular country. It has been applied to dietary NCDs but could and arguably should be adapted to represent the robustness of any public health policy.

## Availability of data and material

All data generated or analysed during this study are included in this published article (and its supplementary information files). Data from government and intergovernmental websites are referenced accordingly in the article.

## Funding

The study was funded by the UK’s Medical Research Council (six countries) (grant number, MR/P025188/1) and EuropAID (grant number, DCI-SANTE/2014/342-479) (Bangladesh). Neither funder played a role in the design, analysis or interpretation of the data.

*Conflict of interest statement.* The authors declare that they have no competing interests. The views are those of the authors alone and do not necessarily reflect the positions of their employing organizations.

*Ethical approval.* Ethical clearance was obtained from University College London and the ethics boards of all participating institutions or national ethics boards (as appropriate) in all six countries, but no human participants were involved in this study.

## Footnotes

1 Nepal has recently moved towards a federal approach in the health sector, but policy-making has not yet been decentralized in the same way as in Pakistan.

2 In the sense of assuming that national policy would be based on and reflect the best available evidence (and in this case WHO recommendations based on that evidence).
